# The quizzical failure of a nudge on academic integrity education: a randomized controlled trial

**DOI:** 10.1186/s41073-023-00139-z

**Published:** 2023-11-30

**Authors:** Aurélien Allard, Anna Catharina Vieira Armond, Mads Paludan Goddiksen, Mikkel Willum Johansen, Hillar Loor, Céline Schöpfer, Orsolya Varga, Christine Clavien

**Affiliations:** 1 iEH2-Institute for Ethics History Humanities, University of Geneva, Geneva, Switzerland; 2 Ottawa Hospital Research Institute, Ottawa, Canada; 3 Department of Food and Resource Economics, University of Copenhagen, Copenhagen, Denmark; 4 Department of Science Education, University of Copenhagen, Copenhagen, Denmark; 5 imCode Partner AB, Visby, Sweden; 6 Department of Public Health and Epidemiology, Faculty of Medicine, University of Debrecen, Debrecen, Hungary

**Keywords:** Moral education, Academic integrity, Nudges, Behavioural insights

## Abstract

**Background:**

Studies on academic integrity reveal high rates of plagiarism and cheating among students. We have developed an online teaching tool, *Integrity Games* (https://integgame.eu/), that uses serious games to teach academic integrity. In this paper, we test the impact of a soft intervention – a short quiz – that was added to the *Integrity Games* website to increase users’ interest in learning about integrity. Based on general principles of behavioral science, our quiz highlighted the intricacy of integrity issues, generated social comparisons, and produced personalized advice. We expected that these interventions would create a need for knowledge and encourage participants to spend more time on the website.

**Methods:**

In a randomized controlled trial involving *N* = 405 students from Switzerland and France, half of the users had to take a short quiz before playing the serious games, while the other half could directly play the games. We measured how much time they spent playing the games, and, in a post-experimental survey, we measured their desire to learn about integrity issues and their understanding of integrity issues.

**Results:**

Contrary to our expectations, the quiz had a negative impact on time spent playing the serious games. Moreover, the quiz did not increase participants' desire to learn about integrity issues or their overall understanding of the topic.

**Conclusions:**

Our quiz did not have any measurable impact on curiosity or understanding of integrity issues, and may have had a negative impact on time spent on the *Integrity games* website. Our results highlight the difficulty of implementing behavioral insights in a real-world setting.

**Trial registration:**

The study was preregistered at https://osf.io/73xty.

**Supplementary Information:**

The online version contains supplementary material available at 10.1186/s41073-023-00139-z.

## Background

Studies on Academic integrity reveal high rates of plagiarism and cheating among students, with about two-thirds of American undergraduates confessing to having plagiarized texts or having cheated during an exam [1, 2]. While some of these acts likely stem from students who knowingly disregard the rules, many questionable practices may be due to ignorance of good academic practice. Indeed, surveys suggest that students often remain confused about the boundaries of what constitutes plagiarism, or what is involved in the proper management of data [3–5].

To improve learning of academic integrity issues, we have developed *Integrity Games*, a new online platform containing serious games for teaching integrity (https://integgame.eu/). While playing the games, users must take on the role of students conducting their own projects and face realistic dilemmas about academic integrity issues. They are asked to choose between various options and their decisions lead to further dilemmas. The website includes four possible cases involving dilemmas related to plagiarism, cooperation between students, and data management (both for qualitative and quantitative data). *Integrity Games* is freely available and accessible in several languages (English, Danish, French, Hungarian, and Portuguese). The website is meant to be included as a module in an independent integrity course, in order to stimulate discussions during class. Completing the four different cases proposed on the website takes about 20 min.

The evaluation of this teaching tool is reported in a companion article [6]. In this article, we report the results of the evaluation of a quiz that we added to the *Integrity Games* website with the goal of stimulating students' curiosity about integrity issues and motivating them to play the *Integrity Games*. The quiz is proposed on the front page of the *Integrity Games* website as an introductory step before playing the games. The design of the quiz was inspired by key results from social and cognitive psychology. First, research on the "pre-testing effect" has shown that asking questions before the learning experience improves retention of the material [7]. Second, our quiz was designed to show the individual relevance (“I will be facing integrity issues”) and the complexity of integrity issues (“It is not easy to make the right choice”). These features aimed at creating a need for knowledge and at highlighting the importance of learning more about integrity issues [8–10]. Third, our quiz highlighted social comparisons and compared students' answers with those of previous users, thus enhancing the social value of integrity issues [11]. Fourth, based on the answers provided in the quiz, the program produced personalized recommendations regarding the most relevant serious games for the user. Pre-testing, highlighting relevance, providing social comparisons, and personalizing the learning experience are four principles that are well-grounded in the behavioral science literature [12]. Our quiz was thus designed to function as a kind of nudge, steering participants towards spending more time on the website without actually constraining them to do so, and without inflicting significant costs [13].

Nudges have been used extensively in the past ten years to improve educational outcomes [14, 15]. Studies have tried a variety of educational nudges, including reminder nudges, commitment nudges, and framing nudges. Reminder studies have tried to motivate students by sending them messages throughout the semester [16–18]. Commitment nudges have instructed students to set goals early in the semester, in order to improve student planning [17, 19]. Framing nudges have manipulated grading presentation for exams to increase student motivation to avoid maluses [20, 21]. Overall, while nudges have been successful in many domains outside of education [22], educational nudges have met with mixed results. While some studies did find positive effects, a few recent large-scale studies of messaging nudges and commitment nudges find little or no impact [14, 17–19]. Despite this mixed record, it is still important to know more about the efficacy of different kinds of nudges. Nudges do not represent a homogeneous category [23], and it is likely that some nudges could work in an educational setting, even if other nudges show little or no effect.

In order to assess whether the quiz has the intended impact on users’ behavior, we designed a randomized controlled experiment. Our aim was to test whether taking the quiz before exploring the serious games would lead students to feel more curious about integrity issues, thus causing them to spend more time on the integrity website, and thus leading them to improve their knowledge of integrity issues.

## Methods

Our study was preregistered on the Open Science Framework platform [24], and received ethical approval from the University of Geneva's Committee for Ethical Research (CUREG-2021–05-57; decision date: 2021–07-05). All materials, data, and code for this study can be found on the Open Science Framework [25].

### Participants

We contacted student organizations at French-speaking Swiss and French universities, asking them to send our survey to their members. We first recruited participants from the five French-speaking Swiss universities, and then sampled French universities, starting with the universities with the largest number of students. We ended the data collection once our preregistered sample size of 400 students was reached. In total, we contacted 190 student organizations based at 17 different universities (5 Swiss, 12 French). We do not have the ability to estimate how many students saw the email offer, since we do not know which student organization chose to forward our email to their members. Participants were paid 10 CHF to complete the questionnaire and to spend at least 10 min on the *Integrity Games* website. We estimated that the full task of playing the games and completing the questionnaire would take between 20 and 30 min.

### Interventions

#### Procedures

Our email to student organizations contained a link to a *LimeSurvey* questionnaire (see Additional file 1, Appendix C, for the exact wording of the questionnaire). After the information and consent phase, all participants answered a short questionnaire aimed at measuring interest regarding integrity issues and provided some socio-demographic information (age, gender, country, study discipline, education level). We also asked students whether their field involved quantitative methods in order to personalize the questions asked later in the survey. Participants were then instructed to explore the *Integrity Games* website for at least 10 min. They were randomly divided into two groups: the intervention group was redirected to a version of the website that required taking the quiz before playing the games, and the control group was redirected to a version of the website that did not include any quiz. The *Integrity Games* website automatically recorded time spent on each page by each participant. Thereafter, all participants answered a second questionnaire, which included the same questions measuring interest regarding integrity issues, additional questions measuring participants’ understanding of integrity issues, and a series of questions measuring how relevant they found the online dilemmas to be. The *Limesurvey* questionnaire was written in French, whereas the quiz and the *Integrity Games* cases were available in both French and English.

#### Description of the quiz

*Integrity Games* presents gamified cases (serious games) addressing a variety of topics on academic integrity: data management, plagiarism, and academic cooperation (6, and for a similar approach, see [26]). Each case confronts students with three successive dilemmas, where students have to make trade-offs between scientific requirements, external pressures and personal advantage. We designed our quiz based on a simple assumption: students are more likely to be motivated to learn about a new topic if they feel that it will help them solve problems in the future. A first major obstacle that could lower students’ motivation to learn about integrity is overconfidence, which would lead students to feel that they don’t have much to learn. A second obstacle is the feeling that integrity issues seldom happen. Our different nudges aim at making students realize how much they have to learn about the topic, and how important this topic will be for their future studies.

**Fig. 1:**
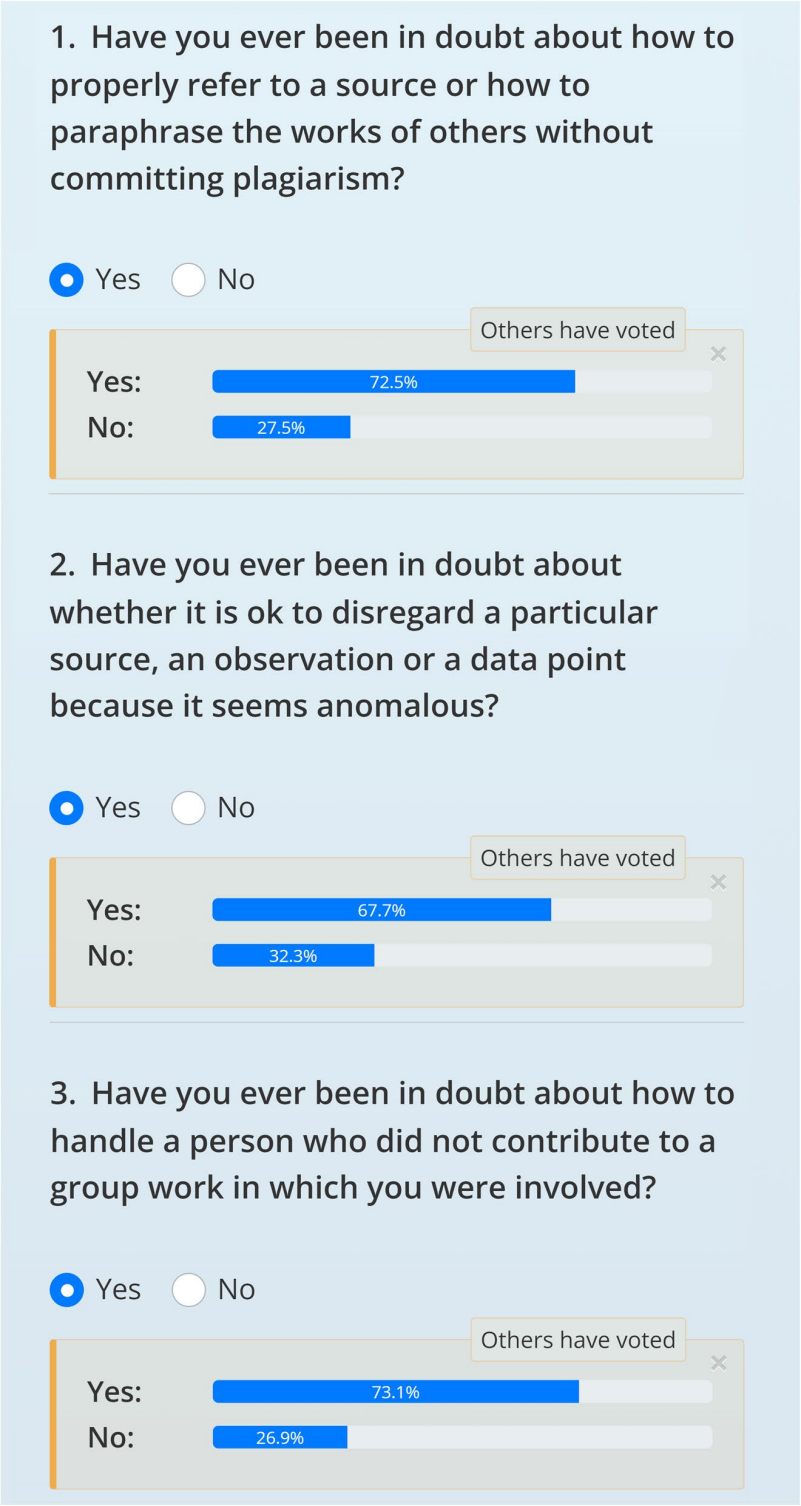
Screenshot of the first page of the quiz on the Integrity Games website. The “Others have voted” boxes only appear after participants have answered all questions. All questions of the quiz can be found in Appendix B

A button for starting the quiz is located on the front page of the *Integrity Games* website. Completing the quiz enables the user to start playing the *Integrity Games*. The quiz includes six questions, which together constitute our nudge (see Additional file 1, Appendix B, for the full text of the quiz, and Fig. 1 to see how our quiz appears on the website). The first three questions ask students whether they have ever been in doubt about plagiarism, data management, or free-riding in collaborative work. For instance, one of the three questions asks: “Have you ever been in doubt about how to handle a person who did not contribute to a group work in which you were involved?” These questions aim at making students realize that integrity issues are frequently encountered in their daily life.

Following the users’ answers to the first three questions, the program automatically outputs information about how other users of *Integrity Games* have answered the same questions. Since most people do in fact encounter such integrity issues, users can realize by seeing the statistics that their peers also regularly encounter similar integrity issues. We hoped that seeing the prevalence of integrity issues would stimulate curiosity and lead students to spend more time on the website by two mechanisms. First, students should realize that, if their peers frequently encounter integrity issues, then they should also face similar difficulties in the future. Second, in a context where integrity issues are widespread, it is useful to learn about academic integrity in order to be able to share this knowledge with others.

The last three questions of the quiz are designed to elicit a need for knowledge by creating a feeling of uncertainty or insecurity. These questions take the form of dilemma questions, presenting fictional cases about integrity issues. For instance, one of the questions asks: “Imagine that you have to write an assignment about a topic you feel unsure about. The assignment is individual and must be passed in order for you to complete the final exam. An older student who is very competent on the topic offers to help you with the assignment. Would you accept?” Users are then asked to choose between the following three options: “Yes”, “No”, “I would hesitate”. The cases present grey zone cases for which clear-cut answers are rarely appropriate, thus prompting users to answer “I would hesitate”. This feature is designed to create a feeling of epistemic insecurity and elicit a need for knowledge.

Finally, once the user has finished answering the questions, the program highlights the integrity cases available on the website that are most relevant for the user. The program selects the games related to the topics that the user has encountered or where the user indicated that they would hesitate if faced with this dilemma. The aim here is to prompt a feeling of personalization, making it clear that taking these dilemmas would lead the student to make better decisions if they encounter these integrity issues in the future.

### Outcomes

To measure participants’ interest in academic integrity, we used four questions allowing for answers on a 5-point scale ranging from “Totally disagree” to “Totally agree”. The full text of the probes can be found in Table 1. We then constituted a *Motivation to learn about Academic Integrity Score* by averaging responses to these questions. Agreement was recoded as a 1 to 5 numeric answer, 5 indicating the highest motivation to learn more about academic integrity. We had not initially planned to validate the scale, as we thought that the questions were straightforward enough to be considered as a face-valid measure of student motivation. However, following one of our reviewer’s comments, we decided to assess the predictive validity of the scale by measuring whether a higher *Motivation to learn about Academic Integrity* score predicts spending more time on the *Integrity Games* website. We report our validation assessment as an exploratory analysis.

**Table 1:** Full probes of the relevance of integrity games scale and motivation to learn about academic integrity score

	**To what extent do you agree with the following statements:**
	**Relevance of Integrity Games Score**
**1**	I have already experienced some situations described in Integrity Games
**2**	The situations described in the cases often happen to students like me
**3**	I found that the cases were especially relevant for student life
**4**	It was nice to play the dilemmas in Integrity Games
**5**	I recommend the Integrity Games website to teachers who are preparing classes on academic integrity for Bachelor students
	**Motivation to learn about Academic Integrity Score**
**1**	I think that participating in courses on academic integrity could be useful to me
**2**	I think that learning about academic integrity is not important for my future studies. [Reversed]
**3**	I think that learning about academic integrity is important
**4**	I would not like to have more teaching on academic integrity. [Reversed]

To measure whether students found the *Integrity Games* to be relevant and interesting, we used five questions allowing for answers on a 5-point scale ranging from “Totally disagree” to “Totally agree”. The full text of the probes can be found in Table 1. We then constituted a *Relevance of Integrity Games Score* by averaging participants’ answers on these five questions.

To measure participants’ understanding of integrity issues, we used nine questions presenting different integrity issues. Participants had to indicate for each issue whether it constituted a violation of scientific integrity by choosing among the following five options: “Yes, it is a clear violation of scientific integrity”, “It is probably a violation of scientific integrity”, “It depends on the situation”, “It is probably not a violation of scientific integrity”, “No, it is clearly not a violation of scientific integrity”.

We designed the scale to measure global understanding of integrity issues. For each question, some response options were clearly unjustifiable (see Table 2). We coded these unjustifiable choices as 0 and coded the remaining options as 1. Our *Understanding of integrity issues* scale includes two different kinds of questions: questions for which the most justifiable answers are on one side of the scale, and questions for which extreme answers on the scale are unjustifiable (e.g., “It’s a clear violation of research integrity” is false). For instance, we consider that “Copying one full page from an external source into your own assignment while marking it as a quote (with a reference to the source).” is generally not a violation of research integrity, so that the correct answers are “It is probably not a violation of scientific integrity” or “No, it is clearly not a violation of scientific integrity”. On the other hand, other questions are much more context-dependent. “Using original ideas provided by a friend in an individual assignment without mentioning the friend’s contribution” can be acceptable in some contexts, for instance if the original ideas comprise a small part of the assignments, and can be a violation of academic integrity in other contexts, for instance if the ideas are used without the friend’s knowledge and if the friend planned to use the ideas for the same assignment. We thus considered the three moderate options to be justifiable: “It is probably a violation of scientific integrity”, “It depends on the situation”, “It is probably not a violation of scientific integrity”. However, we considered that it was unjustifiable to claim that using ideas from a friend was always a violation of academic integrity, or that it was never a violation of academic integrity. We calculated participants’ overall understanding score by computing the mean number of correct answers on the full scale.

**Table 2:** Full probes of the understanding of academic integrity scale

	**Please indicate whether the following actions would constitute a violation of scientific integrity:**
	**Questions for which the correct answer is “Yes, it is a clear violation of scientific integrity”, or “It is probably a violation of scientific integrity”**
**1**	Copying one full page from an external source into your own assignment without marking it as a quote, but including a reference to the source
**2**	Incorporating a part of an assignment that you have previously handed in for another course, into a second assignment that you are about to submit, without making any reference to the first one
**3**	Including a paragraph written by a family member in an exam assignment submitted in (only) your name without mentioning the other person’s contribution
**4**	Not mentioning a relevant source [For students in quantitative fields: During a statistical analysis, deleting a data point] because it goes against your hypothesis
	**Questions for which the correct answer is “No, it is clearly not a violation of scientific integrity” or “It is probably not a violation of scientific integrity”**
**5**	Copying one full page from an external source into your own assignment while marking it as a quote (with a reference to the source)
	**Questions for which the three middle, moderate answers are correct: “It is probably a violation of scientific integrity”, “It depends on the situation”, “It is probably not a violation of scientific integrity”**
**6**	Using original ideas provided by a friend in an individual assignment without mentioning the friend’s contribution
**7**	Adding the name of a group member who contributed much less than the rest of the group to the list of authors of a group assignment
**8**	Not mentioning a source because you think it is not reliable. [For students in quantitative fields: During a statistical analysis, deleting a data point because it seems anomalous]
**9**	Quoting an informant or a source from memory. [For students in quantitative fields: During a statistical analysis, replacing a missing data point by its most likely value]

The division of questions into items for which positive, negative or moderate answers are justifiable was done a priori and was recorded in our preregistration. However, after we collected our data, we realized that our 9^th^ question was ambiguous. We indicated that the moderate answers for the question “During a statistical analysis, replacing a missing data point by its most likely value” were the only justifiable, because we had in mind the possibility of using techniques such as mean imputation, or multiple imputation, that are commonly used in scientific research to deal with missing data [27]. However, we now feel that students would probably not be aware of this possibility, and thus may have thought that the action exclusively referred to unjustifiable data fabrication. In our results section, we report the analysis of the *Understanding of integrity issues* score both with and without the inclusion of the 9^th^ question.

Finally, to measure participant engagement with the serious games, we report both the number of pages that participants visited on the *Integrity Games* website and the total time participants spent on the website. We considered a page as “visited” when a participant spent at least 5 s on the page.

### Sample size

The sample size was determined a priori based on available funds. We only had CHF 4,000 available to pay participants.

### Statistical methods

For all our analyses, we use two-sided tests with an alpha set at 0.05. We control for multiple testing with the Benjamini–Hochberg false-discovery rate correction for all main five statistical tests [28].

Throughout the article, we identify two main effect sizes of interest: changes between the start and the end of the experiment, on the one hand, and comparisons between the different experimental conditions, measured after the experiment, on the other hand. In the case of the identification of changes between the start and the end of the experiment, we collapse across conditions and use a within-subject t-test. In the case of comparisons between our two experimental conditions, we have a pre-experimental measure for one variable (*Motivation to learn about Academic Integrity*) and only possess post-experimental measures for other variables (*Understanding of Academic Integrity* scale, time spent on the website). In the case of *Motivation to learn about Academic Integrity*, we use a linear regression to predict final motivation to learn more about research integrity, based on experimental condition, and controlling for initial level of motivation. In the case of variables for which we only have post-experimental measures, we compare the two experimental conditions using a t-test.

In the exploratory validation of our *Motivation to learn about Academic Integrity* scale, we use a linear regression to predict time spent on the *Integrity Games* website based on *Motivation to learn about Academic Integrity* and experimental condition. To match time spent on the Integrity Games website with our *Motivation to learn about Academic Integrity* scale, we use time spent on the *LimeSurvey* question requiring participants to explore the *Integrity Games* website as a proxy for actual time spent on the website.

We do not use time spent on this specific *LimeSurvey* question in our main analysis comparing time spent on the *Integrity Games* website between the two experimental conditions. In this case, we have a better measure of time spent on the *Integrity Games* website, that is, time logs recorded directly from the *Integrity Games* website. However, since we could not match participants from the *LimeSurvey* questionnaire with participants on the *Integrity Games* websites, we have to use time spent on the *LimeSurvey* question as a proxy in order to validate our scale. Time spent on this question is arguably a noisy measure of engagement with academic integrity. This is due to the fact that it is a compound of actual time spent on the website with breaks that students may have made to visit other websites. However, while not a perfect measure, time spent on this question still serves as a useful measure of actual engagement with integrity issues.

In this analysis, contrary to our other models, we choose not to exclude participants who have spent less than 5 min on the *Integrity Games* website. This is due to the fact that restricting the range of the dependent variable can have a strong downward bias on the estimation of effects. However, we do exclude participants who did not finish the survey, were not students, and showed signs of inattention (as shown with a high Mahalanobis distance; see below for more details). Furthermore, since the dataset contains outliers, we also exclude participants whose time spent on the *Integrity Games* website is more than 3 standard deviations away from the mean.

For exploratory purposes, we also report whether the predictive power of our *Motivation to learn about Academic Integrity* scale changed based on the condition participants were assigned to. In an additional linear regression, we study the interaction between *Motivation to learn about Academic Integrity* and experimental condition, after centering the *Motivation to learn about Academic Integrity* scale to facilitate the interpretation of the *Quiz* coefficient.

Following best practices [29], when we compare two conditions on a numeric variable without covariates, we perform throughout Welch t-test instead of Student t-test.

All analyses are performed within the R statistical environment (Version 4.1.1, [30]), and we use the tidyverse, broom, psych, gtsummary and papaja packages for data cleaning, data analysis, and reporting [31–35].

#### Deviations from preregistration

We deviate from the preregistration in the following ways. First, due to technical difficulties, we used the URL instead of the IP address to identify participants on the *Integrity Games* website (see the *Exclusion of participants* section below for more details). Moreover, we ran a few complementary analyses (explicitly labeled as such in the result section) in addition to those announced in the preregistration. First, we did some robustness checks on the time spent by participants on the *Integrity Games* website. Second, as a robustness check, we removed a question that was possibly misleading from our *Understanding of research integrity* questionnaire (see the *Outcomes* section above for more details). Third, we compared both experimental conditions on how relevant participants found the *Integrity game* website. Fourth, as suggested during the peer-review process, we report Cronbach’s alpha for the different scales used in our analysis. Fifth, we report an exploratory validation of the *Motivation to learn about Academic Integrity* scale.

## Results

### Participant flow and participant characteristics

Five hundred twenty participants started the experiment, and 405 finished the survey (320 French, 80 Swiss, 5 from other countries). Of the 241 participants who passed all quality checks (see below), 56% identified as female, 42% as male, and 2% as neither male nor female. 35% of participants were studying at the Bachelor level, 63% at the Master level, and 1.7% at the Ph.D. level. Participants were, on average, 21.63 years old (SD: 2.20). 54% of participants were enrolled in natural sciences, 14% in social sciences, 11% in humanities, and 21% in other fields including mathematics, medicine, law and architecture (all less than 5% each). Our sample thus incorporates students from a broad range of disciplines. See the Additional file 1, Appendix A, for the full descriptive statistics of our participants.



#### Exclusion of participants

In accordance with our preregistration, we applied three successive exclusion criteria. First, we excluded participants from our analysis who spent less than 5 min on the *Integrity Games* website. This led to the retention of 264 participants.

Second, we excluded participants who reported that they were not students at the time of taking the survey. This led to the retention of 258 participants.

Third, we excluded participants with a Mahalanobis distance higher than the 99% quartile of a Chi-Squared distribution with 8 degrees of freedom based on the four items of the *Motivation to learn about Academic Integrity* scale used at the beginning and at the end of the experiment [36]. We use the Mahalanobis distance to exclude participants due to its simplicity, and due to the fact that a high Mahalanobis distance on these items would be a reliable sign of inattention (for instance, a high Mahalanobis distance could indicate that participants provided divergent answers on items that are highly correlated).

After applying this exclusion criterion, the final number of participants was 241.

To test whether participants in the *Quiz* condition spent more time on the *Integrity Games* website than participants in the *No Quiz* condition, we originally planned to match participants’ IP addresses as recorded on the *Integrity Games* website and participants’ IP addresses as recorded on the *LimeSurvey* website. However, the two websites recorded IP addresses based on different IP protocols (IPv4 vs IPv6), making it impossible to establish a one-to-one match between the two datasets. However, the *Integrity Games* website recorded the referred URL, making it possible for us to infer who participated in the experiment. We included any participant whose referred URL included either “unige” (the university that hosted the *Limesurvey* questionnaire) or “lang = fr”. This led to the identification of 312 participants in the two conditions. While we feel confident that visitors using these two referred URL would have been participants from our experiments, this method did not give us any means of matching participants from the *Limesurvey* dataset and participants from the *Integrity Games* dataset. In particular, this gave us no means of identifying participants who were excluded from the final analyses in the *LimeSurvey* dataset. The subset of analyses that use time spent on the website is thus based on a different subset of participants compared to the other analyses.

### Motivation to learn about academic integrity

We constituted a *Motivation to learn about Academic Integrity* score by averaging the different items of the scale. Cronbach’s alpha was 0.73 for the initial *Motivation* scale, and 0.72 for the final *Motivation* scale. Participants expressed an initial motivation of about 3.8 in both conditions (see Table 3). As preregistered, using a within-subject t-test and merging both experimental conditions, we found that participants’ motivation increased following the exposure to the *Integrity Games* website (${M}_{d}=0.23$, 95% CI $[0.17$, $0.30]$, $t\left(240\right)=6.87$, $p<.001$; *p*-value adjusted for multiple comparisons: < 0.001).

**Table 3:** Descriptive statistics for the main dependent variables, by experimental condition, with mean (SD)

Characteristic	No Quiz, *N* = 131	Quiz, *N* = 110
Understanding of Academic Integrity	0.69 (0.15)	0.69 (0.15)
Motivation to learn about Academic Integrity (Pre-experimental)	3.88 (0.51)	3.78 (0.71)
Motivation to learn about Academic Integrity (Post-experimental)	4.07 (0.62)	4.07 (0.62)
Relevance of the Games	3.96 (0.61)	3.94 (0.62)

As preregistered, to test whether taking the quiz led students to show more interest in learning about academic integrity, we used a linear regression predicting participants’ final score on the *Motivation to learn about Academic Integrity* scale, based on participants’ experimental condition, and controlling for participants’ initial motivation. We found no significant difference between the *Quiz* condition and the *No Quiz* condition ($b=0.06$, 95% CI $[-0.07$, $0.18]$, $t\left(238\right)=0.89$, $p=.372$; *p*-value adjusted for multiple comparisons: *p* = 0.62. See Table 4).

**Table 4:** Predicting final Motivation to learn about Academic Integrity, based on initial motivation and experimental condition

**Predictor**	${\varvec{b}}$	**95% CI**	${\varvec{t}}\left(238\right)$	${\varvec{p}}$
Intercept	4.04	$[3.96$, $4.13]$	96.04	< .001
Quiz	0.06	$[-0.07$, $0.18]$	0.89	.372
Initial motivation (Centered)	0.64	$[0.54$, $0.74]$	12.54	< .001

### Number of pages visited on the website

**Fig. 2:**
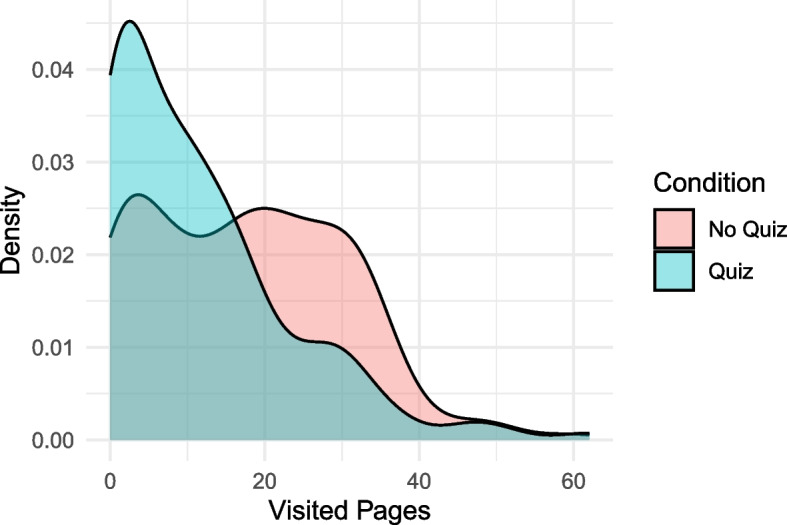
Density plot of the number of visited pages by experimental condition (excluding quiz pages, and after excluding outliers)

Excluding quiz pages, participants visited 17.6 pages of the *Integrity Games* website in the *No Quiz* condition, and 11.5 in the *Quiz* condition (Table 5; Fig. 2). As preregistered, we compared the two conditions with a between-subjects t-test, and found a significant difference ($\Delta M=-5.92$, 95% CI $[-9.56$, $-2.29]$, $t\left(306.30\right)=-3.20$, $p=.001$; *p*-value corrected for multiple comparisons: *p *= 0.004), with participants in the *Quiz* condition visiting about 32% less pages than participants in the *No quiz* condition.

**Table 5:** Number of pages visited and time spent on the Integrity Games website, after excluding outliers. Numbers correspond to mean (SD)

Characteristic	No Quiz, *N* = 165	Quiz, *N* = 147
Visited pages (excluding quiz)	17.62 (12.77)	11.53 (11.84)
Visited pages (incl. quiz)	17.62 (12.77)	14.06 (12.06)
Total time (min.; excl. quiz)	9.33 (8.20)	6.00 (6.55)
Total time (incl. quiz)	9.33 (8.20)	8.58 (7.77)
Cases visited		
0	12%	29%
1	21%	31%
2	27%	20%
3	15%	8.2%
4	25%	12%

### Numbers of pages visited on the website and time spent on the website (Exploratory analyses)

To further investigate the robustness of these results, we ran four additional exploratory analyses. First, we noticed that there were some outliers in both conditions, with some participants visiting about 100 pages. We therefore ran our model again after having excluded participants whose number of visited pages was three standard deviations away from the mean. Excluding outliers slightly increased the effect size ($\Delta M=-6.09$, 95% CI $[-8.85$, $-3.33]$, $t\left(305.45\right)=-4.34$, $p<.001$). All further analyses are conducted after the exclusion of outliers for the respective dependent variables.

Second, we computed whether participants visited fewer pages in the *Quiz* condition if one included the quiz pages in the total pages visited by participants. We find that participants in the *Quiz* condition visited fewer pages than participants in the *No Quiz* condition ($\Delta M=-3.56$, 95% CI $[-6.35$, $-0.77]$, $t\left(303.64\right)=-2.51$, $p=.013$).

Third, we computed the number of integrity cases visited by participants in both conditions. As can be seen in Table 5, participants in the *Quiz* condition were more likely to consult 0 case, and were much less likely to consult 3 or 4 cases. The difference in the proportion of participants who did not visit any case at all is significant (χ^2(1,*n* = 312) = 13.11, *p* < 0.001).

Fourth, we analysed the total time spent on the website, including both quiz and non-quiz questions. Participants spent on average 9.3 min in the *No Quiz* condition, and 8.6 min in the *Quiz* condition; the difference was not significant ($\Delta M=-0.75$, 95% CI $[-2.55$, $1.04]$, $t\left(303.52\right)=-0.83$, $p=.410$). Furthermore, there was no significant difference between the two conditions in terms of the mean number of participants who spent less than a minute on the website (18% in the *Quiz* condition vs 22% in the *No Quiz* condition; χ^2 (1,*n* = 312) = 0.05, *p* = 0.825), indicating that there was not a greater drop-out rate in the *Quiz* condition compared to the *No Quiz* condition.

### Understanding of academic integrity

We constituted an *Understanding of Academic Integrity* score by averaging the different scores on the individual questions, with unjustifiable answers coded as 0 and correct answers coded as 1. Since the *Understanding of Academic Integrity* score was two-dimensional, including both questions for which the correct answer is that a behavior is generally wrong (or, depending on the item, generally correct), and questions for which the correct answer is context-dependent, we analysed Cronbach’s alpha separately for each subscale, and then analysed Cronbach’s alpha for the combined, global scale. Cronbach’s alpha was 0.18 for the subscale where extreme answers are correct, and 0.38 for the subscale where moderate, context-dependent answers are correct. Cronbach’s alpha for the whole scale was 0.07. We proceeded with the analysis of the whole scale, as specified in our preregistration (but see the *Discussion* section for an analysis of the low reliability of the scale).

Participants answered correctly 69% of questions related to the understanding of academic integrity issues in the *No Quiz* condition, and 69% in the *Quiz* condition. Participants would on average get 49% of correct answers if they had chosen to answer randomly. As preregistered, we used a between-subject t-test to compare both conditions. We did not find that the *Quiz* condition had a significant impact on participants’ understanding of integrity (ΔM = 0.00, 95% CI [- 0.04, 0.04], t(231.00) =—0.03, *p* = 0.972; corrected p-value for multiple comparisons: 0.972).

As noted in the *Materials and Procedures* section, one question of the *Understanding of Academic Integrity* scale could be seen as misleading. We therefore ran an additional, non-preregistered analysis. While excluding this question slightly increased the average score, which went from 0.69 to 0.71 across both conditions, it did not change the lack of significant difference between the *Quiz* and the *No Quiz* conditions ($\Delta M=-0.01$, 95% CI $[-0.05$, $0.03]$, $t\left(234.22\right)=-0.55$, $p=.580$).

### Relevance of integrity games

Across conditions, the *Relevance of the Games* score was 3.95 on a 1 to 5 scale. In a non-preregistered analysis, using a between-subject t-test, we found no significant difference between the *Quiz* and the *No Quiz* conditions on the *Relevance of the Games* score ($\Delta M=-0.02$, 95% CI $[-0.18$, $0.13]$, $t\left(231.11\right)=-0.28$, $p=.784$; *p*-value corrected for multiple comparison: 0.784).

### Exploratory validation of the motivation to learn about academic integrity scale

In an exploratory validation of the *Motivation to learn about Academic Integrity* scale, we use a linear regression to predict time spent on the *Integrity Games* website based on *Motivation* and experimental condition. Higher score on the *Motivation* scale significantly predicted spending more time on the Integrity Games website ($b=1.98$, 95% CI $\left[0.72,3.23\right]$, $t\left(366\right)=3.10$, $p=.002$; Table 6).

**Table 6:** Predicting time spent on the Integrity Games website, based on initial motivation to learn about academic integrity and experimental condition. Time is indicated in minutes

Predictor	$b$	95% CI	$t$	$df$	$p$
Intercept	9.32	[8.26, 10.37]	17.39	366	< .001
Motivation about integrity	1.98	[0.72, 3.23]	3.10	366	.002
Quiz	-0.23	[-1.81, 1.34]	-0.29	366	.773

In a further exploratory model, we study whether the predictive power of our *Motivation to learn about Academic Integrity* scale changed based on the condition participants were assigned to. In an additional linear regression, we study the interaction between *Motivation to learn about Academic Integrity* scale and experimental condition. The interaction term was significant, and negative ($b=-2.59$, 95% CI $\left[-5.09,-0.09\right]$, $t\left(365\right)=-2.04$, $p=.042$. See Additional file 1, Table S1 in Appendix E). Since the *Quiz* condition was coded as 1 and the *No Quiz* condition as 0, this coefficient indicates a lower impact of *Motivation* in the *Quiz* condition.

## Discussion

In this experiment, we implemented commonly acknowledged principles of behavioral sciences into an educational nudge. We developed a quiz with the aim to create a need for knowledge, to generate a feeling of social relevance by using peer comparisons, and to increase motivation by providing personalized advice. However, our quiz did not increase interest or understanding of academic integrity issues, and had no impact on the total time spent on the website. In fact, it led participants to spend less time playing the *Integrity Games*.

Even though it was not the main goal of this study, we also measured the overall impact of the *Integrity Games* website on student motivation. We found an increase in *Motivation to learn about Academic Integrity* between the start and the end of the experiment, which can be interpreted as a positive impact of the *Integrity Games* website. However, this is a pure pre / post design, and does not include a control group, so this result should be interpreted with caution. Moreover, we fail to find such an increase in motivation in a companion paper studying the impact of the *Integrity Games* website (without the quiz) [6]. Further research should investigate possible causes for this discrepancy.

Several explanations can account for the failure of the quiz to increase the motivation of our participants. First, the quiz highlighted the complexity of integrity issues. It is possible that stressing this difficulty led participants to be discouraged about the topic. Second, the personalized feedback may have led participants to focus on the sole dilemmas that were recommended to them at the end of the quiz. This could explain why participants in the *Quiz* condition hardly ever visited three or four cases. Personalized feedback may not be appropriate if the goal is to stimulate general interest in a given topic.

### Limitations

A first limitation of our design is the fact that, due to rejection of participants from analysis, we ended up with a relatively low sample size (N_analysed_ = 241). This could lead to a lack of statistical power to detect small positive differences in favor of our nudge. However, the 95% confidence intervals generally do not include any value that could be interpreted as strong benefits of the quiz. For instance, in the *Motivation to learn about academic integrity* scale, the 95% confidence interval allows us to reject any difference stronger than 0.18 (on a 1 to 5 scale) in favor of the *Quiz* condition. Moreover, in terms of time spent on the *Integrity Games* website, we do find a statistically significant difference, but in the opposite direction compared to what we had predicted, with people spending less time on the website in the *Quiz* condition. Overall, the relatively low sample size does not prevent us from inferring a lack of superiority of the *Quiz* condition compared to the *No Quiz* condition.

Second, while the *Motivation to learn about academic integrity* scale had adequate reliability, the *Understanding of academic integrity* score had very low reliability. This low reliability should not be seen as particularly surprising, since the scale incorporated into a global score different aspects of moral judgment, including both a sensitivity to cases where a behavior should be seen as clearly wrong, and a sensitivity to grey zones where context-dependent answers were correct. Furthermore, it incorporated many dimensions of academic integrity, such as integrity in data analysis, cooperation, and honesty in acknowledging sources. While the low reliability could have been expected, it does raise the issue of whether there is a single psychological variable corresponding to integrity knowledge that could be captured in our experiment. It could also be the case that our questions were too complex and led to misunderstandings among students.

A third limitation concerns the fact that our experimental design differed from the normal way *Integrity Games* will be used. *Integrity Games* is intended to be used as a complement to a class teaching research integrity, while our participants took it on their own as part of a research survey. Furthermore, students were paid to participate in our experiment. Psychological literature suggests that an extrinsic motivation to perform a task for a monetary reward may crowd out intrinsic motivation [37]. However, while crowding out by monetary incentives could have affected the behavior of participants, we would expect this to affect in equal measure the *Quiz* and the *No Quiz* conditions. If students had a lower level of intrinsic motivation to learn about integrity due to the monetary compensation, it should have affected them in both conditions. The payment of compensation would only have biased our results if there was an interaction between financial compensation and quiz taking. We think that such an interaction is unlikely.

On the other hand, it could be argued that our students were already too motivated, thus leading to a ceiling effect. This worry is substantiated by the fact that students showed on average a high level of motivation, with initial levels of *Motivation to learn about Academic Integrity* around 3.8 on a 1 to 5 scale. Furthermore, in an exploratory model predicting time on the *Integrity Games* website, we found a significant interaction between experimental condition and *Motivation to learn about Academic Integrity*, with *Motivation to learn about Academic Integrity* having significantly less predictive power in the *Quiz* condition compared to the *No Quiz* condition. If this exploratory result holds in further replications, it would mean that the *Quiz* condition had a negative impact on students with a high *Motivation to learn about Academic Integrity* score, and a positive impact on students already low on *Motivation to learn about Academic Integrity*. This result would make sense, as students who are motivated to learn more about academic integrity already know that they do not know enough about the topic, so our quiz could be a waste of time for them, and could discourage them from spending more time on the *Integrity Games* website. The positive impact of the quiz would then only concern students with a low level of motivation, who need to be motivated by realizing how much they don’t know. However, since this is an exploratory result, further research should substantiate whether the quiz indeed has a differential impact on students based on their initial level of motivation.

A fourth potential limitation is that we did not use a validated scale to measure motivation to learn about academic integrity, but built our own scale for that purpose. However, we believe that the scale performed well. First, Cronbach’s alpha was above 0.7, indicating adequate reliability. Second, in an exploratory model, our scale showed good predictive validity. An increase of 1 point on our scale predicted spending 2 more minutes on the *Integrity Games* website. Participants who reported being motivated thus evinced authentic curiosity to learn more about integrity issues.

At last, a sixth possible limitation concerns our own conflict of interest: we are both the creators of the *Integrity Games* website and the designers of the quiz. However, this conflict of interest would have given us additional incentives to find a positive impact of the quiz on participants’ motivation. Contrary to our hopes, we found disappointing results regarding the impact of the quiz.

### Future directions

Given the mixed results that we found for our quiz, we would like to highlight three important directions for future research.

First, our quiz is similar in spirit to tests illustrating the pre-testing effect, or the effect showing that asking questions to participants before they learn a material can lead to better retention of the material. However, contrary to typical pre-testing effect experiments, we do not provide correct answers to participants. Our goal was to raise curiosity by leaving students uncertain about the true answer, thus creating a feeling that more knowledge is needed. However, it is possible that curiosity is better produced by showing to students that they are wrong, rather than highlighting that they don't know. It is possible that knowledge of error is more motivating than awareness of uncertainty. Future research should study whether highlighting students' mistakes could lead to increased motivation to learn academic integrity.

A second promising new line of research would be to study how the quiz can be used in different academic populations. Our exploratory result on the differential impact of the quiz on students with different levels of motivation suggests that the quiz may be more efficient for students who are initially unmotivated to learn more about academic integrity. Future research could replicate our work to see if this effect is indeed robust.

A third interesting direction of research would be to embed the quiz within a more realistic teaching environment. Our quiz was online, and recruited students who were not currently taking integrity classes. In a more realistic setting, a teacher could reinforce the uncertainty created by the quiz by highlighting the intricacy of academic integrity issues. Furthermore, the quiz includes aspects of social comparisons, which may be more salient if the student has actual peers they can compare themselves to.

## Conclusion

In a randomized experiment, we have implemented a nudge, in the form of a short quiz, designed to raise curiosity about research integrity and promote further learning. However, we found that the nudge did not lead to higher expressed curiosity, and led students to spend less time on our educational website.

While our experiment raises the issue of the difficulty of implementing behavioral insights, it does not necessarily imply that the use of personalized recommendations, or the importance of creating a need for knowledge, are bad practices. More research is needed to understand how behavioral insights can be translated into effective educational practices.

The last 20 years have seen the development of practical applications of behavioral sciences to increase prosocial actions and environmental choices [12, 13]. However, despite the strong enthusiasm for these approaches, recent studies have shown disappointing results, including both replication failures and doubts about the overall strength of nudging effects [38–40]. Our experiment reinforces this literature, showing the difficulty of implementing general behavioral principles in moral education.

### Supplementary Information

**Additional file 1. **

## Data Availability

All materials, data, and code for this study can be found on the OSF (https://osf.io/jdzwb/).
