# Supplementary material for: The quizzical failure of a nudge on academic integrity education: a randomized controlled trial
Source: Res Integr Peer Rev. 2023 Nov 30;8:15. doi: 10.1186/s41073-023-00139-z (PMC10688455; doi:10.1186/s41073-023-00139-z)
Supplement: Supplementary file 1 — Additional file 1. [file 41073_2023_139_MOESM1_ESM.docx]

**This is a supplementary file to the manuscript: “The quizzical failure of a nudge on academic integrity education: a randomized controlled trial”**

Table of content

[Appendix A Participants baseline characteristics 2](#_Toc146541163)

[Appendix B Content of the quiz 3](#_Toc146541164)

[Appendix C Limesurvey Questions 6](#_Toc146541165)

[Appendix D: Full comparison of success rates for all nine probes on understanding academic integrity 13](#_Toc146541166)

[Appendix E: Exploratory model predicting differential impact of motivation based on experimental condition 17](#_Toc146541167)

# Appendix A Participants baseline characteristics

| **Characteristic** | **No Quiz**, N = 131 | **Quiz**, N = 110 |
| --- | --- | --- |
| Age | 21.48 (1.85) | 21.82 (2.55) |
| Gender |  |  |
| Men | 58 (44%) | 44 (40%) |
| Women | 71 (54%) | 63 (57%) |
| Other | 2 (1.5%) | 3 (2.7%) |
| Country |  |  |
| France | 98 (75%) | 96 (87%) |
| Switzerland | 32 (24%) | 14 (13%) |
| Other | 1 (0.8%) | 0 (0%) |
| Study Level |  |  |
| Undergraduate | 46 (35%) | 38 (35%) |
| Master | 82 (63%) | 71 (65%) |
| Ph.D. | 3 (2.3%) | 1 (0.9%) |
| Study type |  |  |
| Qualitative | 19 (15%) | 16 (15%) |
| Quantitative | 112 (85%) | 94 (85%) |
| Motivation to learn about Academic Integrity (Pre-experimental) | 3.88 (0.51) | 3.78 (0.71) |

# Appendix B Content of the quiz

[On the starting page of the quiz, participants could read the following three questions:]

1. Have you ever been in doubt about how to properly refer to a source or how to paraphrase the works of others without committing plagiarism?
   - - Yes
     - No
2. Have you ever been in doubt about whether it is ok to disregard a particular source, an observation or a data point because it seems anomalous?
   - - Yes
     - No
3. Have you ever been in doubt about how to handle a person who did not contribute to a group work in which you were involved?
   - - Yes
     - No

[After answering the series of questions, the follow text would appear at the bottom of the page:]

How did others answer? Click the button and find out how previous players felt about these three situations. Scientific studies show that most university students experience these sorts of dilemmas in the course of their education. Click the button and watch the diagram under every question!

[Here is an example of the diagram that participants could see:]


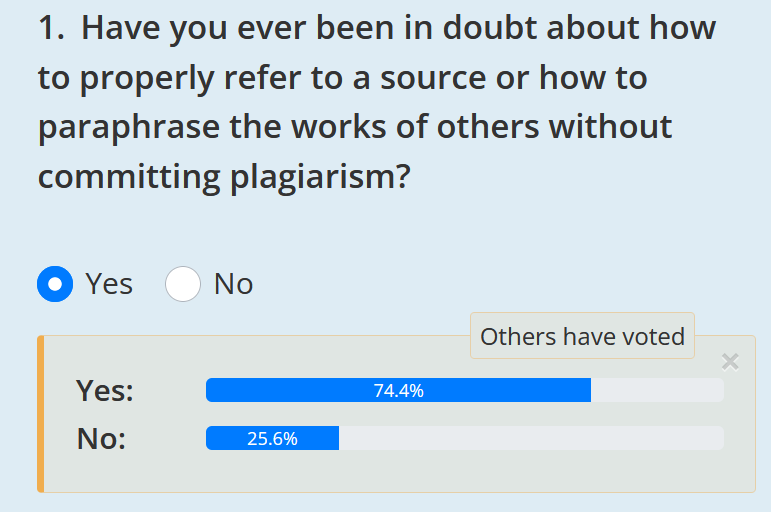


------------------------------------ page break ----------------------------------------

[On the next page, participants could read the following three questions:]

1. Imagine that you discover that one of your best friends has copied several pages from an unpublished master's thesis into a project you are working on together. Even though you point out the plagiarism, your friend does not want to rewrite this part of the text.

Would you report your friend to the teacher?

- Yes
- No
- I would hesitate

1. Imagine you have to hand in an assignment where you have to present an interpretation of some "information" that you have gathered - it could be data from an experiment, transcripts of interviews, historical sources or other literature. You have collected more information than strictly necessary, and are now facing the challenge to keep your assignment within the mandatory word limit. Is it ok, for the sake of simplicity and shortness, to mention and discuss only the best half of the information you collected?

- Yes
- No
- I would hesitate

1. Imagine that you have to write an assignment about a topic you feel unsure about. The assignment is individual and must be passed in order for you to complete the final exam. An older student who is very competent on the topic offers to help you with the assignment. Would you accept?

- Yes
- No
- I would hesitate

[If the participants answered with either “Yes” or “No” to any of the preceding three questions, the following text appeared:] Would you be comfortable if an external observer asked you to justify the choice you made in question [insert relevant question number here]? Can you motivate why [you chose not to report your friend to the teacher? / you chose to mention only half of the information in your essay? / you chose to refuse the student's offer to help you?]

------------------------------------ page break ----------------------------------------

[On the final page, participants could read the following feedback. The exact feedback depended on their previous answers:]

This is our personalized proposal to you. You will always be able to revisit this page, if you click on "My Cases" or "Quiz" in the menu.

Based on your answers to the quiz, the following dilemma(s) may be of particular interest to you:

- The Plagiarism Case
- The Collaboration Case

Select one of the cases depending in your type of work (qualitative or quantitative):

- The Qualitative Data Case
- The Quantitative Data Case

# Appendix C Limesurvey Questions

Thank you for your interest in this study. Our research aims to evaluate the usability and effectiveness of new teaching materials that we are developing as part of the INTEGRITY research project, funded by the European Horizon 2020 project. These teaching materials are online games that will make you read cases of ethical dilemmas commonly encountered by students. You will have to indicate the choices you would make if you were confronted with these dilemmas. The purpose of these games is to help you better understand the issues related to scientific integrity. Your answers will help us to identify potential improvements for teaching integrity in higher education.

You are under no obligation to participate in this study and are free to withdraw at any time. If you follow all the steps of the procedure (estimated time: 20'), we will compensate you for your participation by making a transfer of 10 CHF (about 9.20 euros, if your account is in euros) to your Paypal account. If you do not have a Paypal account, you will receive an email from Paypal inviting you to create your account to receive your payment.

In order to proceed with the transfer of your compensation via Paypal, we will collect your email address. This will be kept exclusively on the computer of the principal researcher, and will be destroyed four weeks after your participation. We will also temporarily collect the IP addresses of the computers. We will delete these IP addresses four weeks after your participation. Apart from these data, no identifying information will be collected.

Since we will be destroying all identifying information in the next few weeks, we will have no way to link your responses to your identity. As a result, once the responses are recorded, we will not be able to destroy them if you request it in more than four weeks. The anonymized data will be kept and archived under the responsibility of the principal investigator, without time limit.

If you would like to learn more about this research or have any questions, you can email us at [email removed]. If you wish, we will be able to send you the preliminary results of the study in early 2022.

On the basis of the above information, I confirm my agreement to participate in this research and authorize the use of the data for scientific purposes and the publication of the results of the research in scientific journals or books, with the understanding that the data will remain anonymous and that no information will be given about my identity.

Please select only one of the following:

Yes

No

------------------------------------ page break ----------------------------------------

Are you a student?

Yes

No

What is your current level of education?

Bachelor's degree or equivalent

Master's degree, or equivalent

Doctorate, or equivalent

How old are you?

In which country do you currently live?

Switzerland

France

Other

In which program of study are you currently enrolled?

Does your field of study involve quantitative methods?

(Quantitative: using statistics, such as the mean, standard deviation,...)

Yes

No

Which gender do you primarily identify with?

Man

Woman

Other

------------------------------- Page break ------------------------------

To what extent do you agree with the following statements:

[Choose the appropriate answer for each item:

Totally disagree

Disagree

Neutral

Agree

Totally agree]

- I think that participating in courses on academic integrity could be useful to me.
- I think learning about academic integrity is not important for my future studies.
- I think learning about academic integrity is important.
- I would not like to have more teaching on academic integrity.

---------------------------------------------- page break -----------------------------------------

[No quiz condition]

STOP!

Keep this window open, but the rest of this experiment will take place here: <https://noq.integgame.eu/?lang=fr>.

Before continuing with the questionnaire, please explore carefully a website we have developed on research integrity. This website is an educational tool with scenarios where you have to make choices about integrity issues (like a game where you are the hero).

Please explore the different scenarios accessible from the home page.

You can change the language to "French" at the top right of the page.

Once you have explored the website for at least 10 minutes, come back to this page and answer the final questions.

I attest that I have spent at least 10 minutes exploring the Integrity Games website.

Yes

No

[Quiz condition]

STOP!

Keep this window open, but the rest of this experiment will take place here: https://integgame.eu/?lang=fr.

Before continuing with the questionnaire, please explore carefully a website we have developed on research integrity. This website is an educational tool with scenarios where you have to make choices about integrity issues (like a game where you are the hero).

Please complete the quiz at the bottom right of the home page, then explore the different scenarios.

(You can change the language to "French" at the top right of the page).

Once you have explored the website for at least 10 minutes, come back to this page and answer the final questions.

I attest that I have spent at least 10 minutes exploring the Integrity Games website.

Yes

No

----------------------------------- page break ------------------------------------

Which scenarios have you explored?

The collaboration case

The plagiarism case

The quantitative research case

The qualitative research case

None of this

To what extent do you agree with the following statements:

Totally disagree

Disagree

Neutral

Agree

Totally agree

- I think that participating in courses on academic integrity could be useful to me.
- I think learning about academic integrity is not important for my future studies.
- I think learning about academic integrity is important.
- I would not like to have more teaching on academic integrity.

To what extent do you agree with the following statements:

Totally disagree

Disagree

Neutral

Agree

Totally agree

- I have already experienced some situations described in *Integrity Games*.
- The situations described in the scenarios often happen to students like me.
- I found that the scenarios were especially relevant for student life.
- It was nice to play the dilemmas in *Integrity Games*.
- I recommend the *Integrity Games* website to teachers who are preparing classes on academic integrity for Bachelor students.

--------------------------------------------------- page break ----------------------------------------------

Please indicate whether the following actions would constitute a violation of scientific integrity:

Choose the appropriate answer for each item:

• Yes, it is a clear violation of scientific integrity

• It is probably a violation of scientific integrity

• It depends on the situation

• It is probably not a violation of scientific integrity

• No, it is clearly not a violation of scientific integrity

[The order in which participants saw the following questions was fully randomized]

- Copying one full page from an external source into your own assignment while marking it as a quote (with a reference to the source)
- Copying one full page from an external source into your own assignment without marking it as a quote, but including a reference to the source.
- Incorporating a part of an assignment that you have previously handed in for another course, into a second assignment that you are about to submit, without making any reference to the first one.
- Including a paragraph written by a family member in an exam assignment submitted in (only) your name without mentioning the other person’s contribution.
- Using original ideas provided by a friend in an individual assignment without mentioning the friend’s contribution.
- Adding the name of a group member who contributed much less than the rest of the group to the list of authors of a group assignment.
- Not mentioning a relevant source [For students in quantitative fields: During a statistical analysis, deleting a data point] because it goes against your hypothesis.
- Not mentioning a source because you think it is not reliable. [For students in quantitative fields: During a statistical analysis, deleting a data point because it seems anomalous]
- Quoting an informant or a source from memory. [For students in quantitative fields: During a statistical analysis, replacing a missing data point by its most likely value]

# Appendix D: Full comparison of success rates for all nine probes on understanding academic integrity

| **Category** | **Probes** | **No Quiz**, N = 131^1^ | **Quiz**, N = 110^1^ | **p-value**^2^ |
| --- | --- | --- | --- | --- |
| Acceptable | Copying one full page from an external source into your own assignment while marking it as a quote (with a reference to the source) | 69% | 69% | >0.9 |
| Violation | Copying one full page from an external source into your own assignment without marking it as a quote, but including a reference to the source. | 89% | 92% | 0.5 |
| Violation | Incorporating a part of an assignment that you have previously handed in for another course, into a second assignment that you are about to submit, without making any reference to the first one. | 43% | 40% | 0.7 |
| Violation | Including a paragraph written by a family member in an exam assignment submitted in (only) your name without mentioning the other person’s contribution. | 71% | 75% | 0.5 |
| Grey zone | Using original ideas provided by a friend in an individual assignment without mentioning the friend’s contribution. | 79% | 66% | 0.023 |
| Grey zone | Adding the name of a group member who contributed much less than the rest of the group to the list of authors of a group assignment. | 77% | 79% | 0.7 |
| Violation | Not mentioning a relevant source [For students in quantitative fields: During a statistical analysis, deleting a data point] because it goes against your hypothesis. | 90% | 87% | 0.5 |
| Grey zone | Not mentioning a source because you think it is not reliable. [For students in quantitative fields: During a statistical analysis, deleting a data point because it seems anomalous] | 56% | 58% | 0.8 |
| Grey zone | Quoting an informant or a source from memory. [For students in quantitative fields: During a statistical analysis, replacing a missing data point by its most likely value] | 46% | 54% | 0.2 |
|  | ^1^n (%) | | | |
|  | ^2^Pearson's Chi-squared test | | | |

| **Characteristic** | **No Quiz**, N = 131^1^ | **Quiz**, N = 110^1^ |
| --- | --- | --- |
| Copying one full page from an external source into your own assignment while marking it as a quote (with a reference to the source) |  |  |
| Clearly not a violation | 38% | 43% |
| Probably not a violation | 31% | 26% |
| It depends | 7.6% | 9.1% |
| Probably a violation | 19% | 16% |
| Clearly a violation | 4.6% | 5.5% |
| Copying one full page from an external source into your own assignment without marking it as a quote, but including a reference to the source. |  |  |
| Clearly not a violation | 0.8% | 0.9% |
| Probably not a violation | 6.1% | 1.8% |
| It depends | 3.8% | 5.5% |
| Probably a violation | 40% | 38% |
| Clearly a violation | 50% | 54% |
| Incorporating a part of an assignment that you have previously handed in for another course, into a second assignment that you are about to submit, without making any reference to the first one. |  |  |
| Clearly not a violation | 7.6% | 15% |
| Probably not a violation | 31% | 20% |
| It depends | 19% | 25% |
| Probably a violation | 27% | 25% |
| Clearly a violation | 15% | 15% |
| Including a paragraph written by a family member in an exam assignment submitted in (only) your name without mentioning the other person’s contribution. |  |  |
| Clearly not a violation | 4.6% | 0.9% |
| Probably not a violation | 9.9% | 12% |
| It depends | 15% | 13% |
| Probably a violation | 35% | 40% |
| Clearly a violation | 36% | 35% |
| Using original ideas provided by a friend in an individual assignment without mentioning the friend’s contribution. |  |  |
| Clearly not a violation | 0.8% | 4.5% |
| Probably not a violation | 15% | 13% |
| It depends | 33% | 16% |
| Probably a violation | 32% | 37% |
| Clearly a violation | 20% | 29% |
| Adding the name of a group member who contributed much less than the rest of the group to the list of authors of a group assignment. |  |  |
| Clearly not a violation | 21% | 19% |
| Probably not a violation | 44% | 41% |
| It depends | 28% | 27% |
| Probably a violation | 5.3% | 11% |
| Clearly a violation | 2.3% | 1.8% |
| Not mentioning a relevant source [For students in quantitative fields: During a statistical analysis, deleting a data point] because it goes against your hypothesis. |  |  |
| Clearly not a violation | 0.8% | 0.9% |
| Probably not a violation | 0% | 3.6% |
| It depends | 9.2% | 8.2% |
| Probably a violation | 21% | 19% |
| Clearly a violation | 69% | 68% |
| Not mentioning a source because you think it is not reliable. [For students in quantitative fields: During a statistical analysis, deleting a data point because it seems anomalous] |  |  |
| Clearly not a violation | 2.3% | 10% |
| Probably not a violation | 11% | 8.2% |
| It depends | 26% | 28% |
| Probably a violation | 19% | 22% |
| Clearly a violation | 41% | 32% |
| Quoting an informant or a source from memory. [For students in quantitative fields: During a statistical analysis, replacing a missing data point by its most likely value] |  |  |
| Clearly not a violation | 6.1% | 9.1% |
| Probably not a violation | 5.3% | 8.2% |
| It depends | 14% | 15% |
| Probably a violation | 27% | 31% |
| Clearly a violation | 48% | 37% |
| ^1^% | | |

# Appendix E: Exploratory model predicting differential impact of motivation based on experimental condition

Table S1.

Predicting time spent on the Integrity Games website, based on initial motivation to learn about integrity and experimental condition, with an interaction term

| Predictor | $b$ | 95% CI | $t$ | $df$ | $p$ |
| --- | --- | --- | --- | --- | --- |
| Intercept | 9.30 | [8.26, 10.35] | 17.44 | 365 | < .001 |
| Motivation about Integrity | 3.27 | [1.51, 5.04] | 3.64 | 365 | < .001 |
| Quiz | -0.23 | [-1.80, 1.34] | -0.29 | 365 | .770 |
| Motivation about Integrity $\times$ Quiz | -2.59 | [-5.09, -0.09] | -2.04 | 365 | .042 |
